# Protein Adsorption in Microengraving Immunoassays

**DOI:** 10.3390/s151026236

**Published:** 2015-10-16

**Authors:** Qing Song

**Affiliations:** Chemical and Biomolecular Engineering, New York University Polytechnic School of Engineering, 6 Metro Tech Center, Brooklyn, NY 11201, USA; E-Mail: qs299@nyu.edu; Tel.: +1-646-182-603-863

**Keywords:** protein adsorption, interface, transport dynamics, transport mechanisms, single cells, protein secretion, microengraving, immunoassay

## Abstract

Microengraving is a novel immunoassay forcharacterizing multiple protein secretions from single cells. During the immunoassay, characteristic diffusion and kinetic time scales $\tau_{D}$ and $\tau_{K}$ determine the time for molecular diffusion of proteins secreted from the activated single lymphocytes and subsequent binding onto the glass slide surface respectively. Our results demonstrate that molecular diffusion plays important roles in the early stage of protein adsorption dynamics which shifts to a kinetic controlled mechanism in the later stage. Similar dynamic pathways are observed for protein adsorption with significantly fast rates and rapid shifts in transport mechanisms when $C_{0}^{*}$ is increased a hundred times from 0.313 to 31.3. Theoretical adsorption isotherms follow the trend of experimentally obtained data. Adsorption isotherms indicate that amount of proteins secreted from individual cells and subsequently captured on a clean glass slide surface increases monotonically with time. Our study directly validates that protein secretion rates can be quantified by the microengraving immunoassay. This will enable us to apply microengraving immunoassays to quantify secretion rates from 10^4^–10^5^ single cells in parallel, screen antigen-specific cells with the highest secretion rate for clonal expansion and quantitatively reveal cellular heterogeneity within a small cell sample.

## 1. Introduction

### 1.1. Scaling Arguments of Protein Adsorption Dynamics in Microengraving Immunoassay

Many cells secrete proteins into their surrounding microenvironment. These secreted proteins play important biological functions within living organisms [1,2] and in other processes [3]. For example, lymphocytes are at the core of adaptive immunity. Activation of T or B lymphocytes through contact with foreign antigens and subsequent secretion of multiple cytokines and immunoglobulins (antibodies) are required for production of an effective immune response [4,5,6,7]. Therefore, the ability to detect multiple proteins is critical to characterize immunological responses to pathogens, allergens, or self-antigens in autoimmune diseases [8,9,10].

The microengraving immunoassay was developed based on the mechanism of ELISA [11,12]. A dense, elastomeric array of wells with subnanoliter volumes (125 pL each) was used to generate printed microarrays of cytokines released by polyclonally and antigen-specifically activated human T cells in our previous study [13]. The microengraving immunoassay is based on intaglio printing, in which a glass slide is temporarily sealed to the array of nanowells to capture proteins secreted by confined cells in both a multiplexed and quantitative manner. This immunoassay enabled identification and recovery of antigen-specific cells with highest secretion rates for clonal expansion [14,15].

Adsorption of proteins onto a glass slide is a dynamic process. The effectiveness of protein adsorptions is influenced by rate of delivery of proteins to sublayer of the glass slide as well as adsorption kinetics. Equilibrium surface concentration Γ_e_ depends on the tendency of proteins adsorbed. The simplest model which adequately describes the gross equilibrium behavior of a wide variety of proteins is the Langmuir isotherm [16,17]:

(1) $${\text{Γ}}_{e}/{\text{Γ}}_{\infty }=\left(k_{on}C_{0}/k_{off}\right)/\left(\left(k_{on}C_{0}/k_{off}\right)+1\right)=\left(C_{0}/K_{D}\right)/\left(1+C_{0}/K_{D}\right)$$

where ${\text{Γ}}_{\infty }$ is the maximum surface concentration, *k_on_* and *k_off_* are associate and dissociate constants respectively, and *K_D_* is defined as the ratio of the dissociate to the associate constants, *i.e.*, *K_D_* = *k_off_* / *k_on_*.

**Figure 1:**
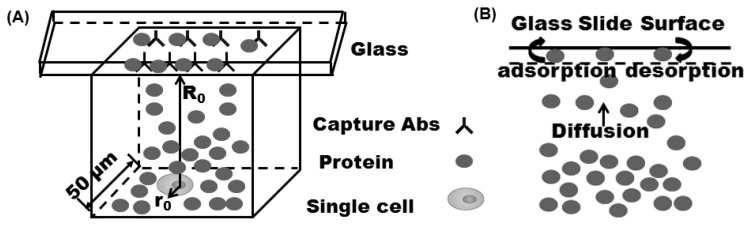
(**A**) Microengraving immunoassay and (**B**) adsorption mechanisms. Once the proteins diffuse to the subsurface they will either instantaneously adsorb at the interface in accordance with the diffusion-controlled model or/and will have to pass through a potential barrier to adsorb (kinetic controlled and diffusive-kinetic-mixed mechanisms).

When proteins are secreted from a single cell in microengraving immunoassay (Figure 1A), they diffuse through microwells to the sublayer of the sealing glass slide and bind onto it. Scaling arguments could provide a quantitative understanding for this process. First, consider the fresh interface adsorption to a planar surface from bulk solution with an initially uniform concentration *C*_0_. At equilibrium, the surface concentration is given by adsorption isotherm Γ_e_ (*C*_0_). The maximum rate at which proteins’ molecular diffusion proceeds is obtained when setting the concentration of proteins at sublayer to zero. Under such circumstance, the surface concentration of proteins absorbed at diffusion controlled limit [16,17,18] is given by:

(2) $${\text{Γ}}_{D}(t)=2C_{0}\sqrt{Dt/\pi }$$

where *τ_D_* defines the characteristic time required for molecular diffusion of proteins through the microwell to establish an equilibrium monolayer assuming infinitely fast surface kinetics. Molecular diffusion time scale is calculated as *τ_D_* = *h*^2^*/D* [16,17], where *h* is the length for the protein’s molecular diffusion. It is constrained by the dimension of the microwell in this study. The kinetic limit at which proteins bind onto the surface of glass slide can be obtained by integrating the kinetic equation by setting the proteins’ sublayer concentration as $C_{0}$ (*i.e.*, no bulk molecular diffusion). The Langmuir equation [16,17,18] of the kinetic adsorption is given by:

(3) $${\text{Γ}}_{K}(t)={\text{Γ}}_{e}\left(1-e^{-(k_{off}+C_{0}k_{on})t}\right)$$

where *τ_K_* is defined as characteristic kinetic time necessary for proteins in the sublayer to bind onto the surface of the glass slide in absence of molecular diffusion. From above equation, the characteristic kinetic time scale *τ_K_* can be estimated as *τ_K_* = (*k_on_C_0_* + *k_off_*)^–1^ by assuming $C_{s}=C_{0}$ [16].

### 1.2. Protein Adsorption Mechanisms in Microengraving Immunoassay

In microengraving immunoassay, when proteins are secreted from a single cell, they first diffuse from the cell surface to a sublayer of the glass slide surface. The sublayer of the glass slide surface is theoretically treated as an imaginary plane (dashed line in Figure 1B), which is a few molecular diameters below the interface. At the initial stage, the surface of the glass slide tends to be an empty site. Then, proteins diffused to the sublayer of the glass slide adsorb directly onto it while also achieving correct orientation (Figure 1B). The glass slide surface becomes occupied with protein adsorption. The probability that proteins will bind to a more crowded site is also increased. Desorption will take effect and back diffusion from the sublayer to the bulk solution must then also be considered. Depending on the rates of diffusion and kinetic association and dissociation, the dynamic protein adsorption process can be limited by (1) molecular diffusion of proteins to the sublayer of glass slide, these cases are referred as diffusion-controlled; (2) surface kinetic adsorption or binding, these cases are defined as kinetic controlled; (3) both steps of diffusion and kinetic adsorption under mixed diffusion-kinetic mechanism.

The diffusion controlled mechanism assumes that molecular diffusion of proteins from the surface of the single cell to a sublayer of the glass slide surface is the rate-controlling step, and the timescale which proteins diffuse from the sublayer to the surface of glass slide is very fast. Under such circumstances, adsorption kinetics are much faster than the molecular diffusion, and protein molecules that reach the sublayer of the glass slide immediately adsorb onto the glass slide surface. The concentration of proteins at the sublayer of the glass slide tends to be zero, and the driving force for the molecular diffusion is simply proportional to the bulk concentration. The larger this concentration, the faster is the transport of proteins from the bulk to the glass slide. Under the diffusion controlled mechanism, the protein surface concentration is proportional to square root of time at the initial stage and inverse-square-root of time for longer time protein adsorption dynamics [19,20].

The kinetic controlled mechanism assumes that the protein adsorption process overall is kinetically controlled and governed by an activation barrier. When proteins reach the sublayer of the glass slide, the proteins do not instantaneously adsorb onto the interface, they need to overcome a potential energy barrier and be in the correct orientation for adsorption. This adsorption barrier decreases the adsorption rate, and hence the transfer of proteins from the sublayer to the interface of glass slide is the rate-determining step [19].

The mixed diffusion-kinetic controlled adsorption assumes that the proteins undergo molecular diffusion from the single cell’s surface to the sublayer of the glass slide, following the same diffusion equations as for the diffusion controlled mechanism. However, kinetic barrier also exerts an influence on the protein adsorption dynamics. This influence requires longer times for protein adsorption to be equilibrated on the glass slide surface than the diffusion controlled cases.

### 1.3. Theoretical Model Method 

To simulate the adsorption dynamics of proteins onto the glass slide surface in microengraving immunoassay, the single cell was assumed to be a sphere of radius r_0_ sitting in the bottom of the microwell (Figure 1A). Convective flow was negligible and molecular diffusion of secreted proteins was predominant in the confined volume. The initial surface concentration of the captured proteins was zero, *i.e.*, Γ=0 at *t* = 0. Protein secretion was treated as a continuous point source defined as N=ϕt on the surface of the single cell [21]. Where *N* is the total proteins secreted from the single cell, ϕ is the rate of secretion, and *t* is the incubation time. The bulk concentration *C*_0_ is defined as protein concentration on the surface of the single cell at a certain incubation time. The dynamic protein transport equations include three equations. The first is the unsteady state diffusion equation in Cartesian coordinates in the microwell:

(4) $$\partial C^{*}/\partial r=\partial {\text{Γ}}^{'}/\partial \tau$$

The second is the kinetic protein adsorption mass balance equation at the glass slide surface. The third is the relation equating the accumulation of proteins on the glass slide surface to the molecular diffusive flux. This correlation matches the kinetic adsorption and molecular diffusive flux at the sublayer of the glass slide. Nondimensionalizing the surface concentration Γ by the maximum surface concentration ${\text{Γ}}_{\infty }$(${\text{Γ}}^{'}=\text{Γ}/{\text{Γ}}_{\infty }$), and time by the diffusion time scale [22] $\tau_{D}$ ($\tau =t/\tau_{D}$), the nondimensional form of kinetic equation [16,17,18] becomes:

(5) $$d{\text{Γ}}^{'}/d\tau =k_{a}\left(C_{R0}^{*}\left(1-{\text{Γ}}^{'}\right)-{\text{Γ}}^{'}\right)$$

where $k_{a}$ is the ratio of the bulk diffusion time scale $\tau_{D}$ to the kinetic scale for dissociation $k_{off}^{-1}$:$k_{a}=k_{off}\tau_{D}$. Note that the diffusional timescale $\tau_{D}$ is selected to nondimensionalize time which is independent of bulk concentrations. Same nondimensional time is used for simulations at different bulk concentrations $C_{0}^{*}$ to represent the same dimensional point in time. Protein concentrations captured on glass slide surface ${\text{Γ}}^{'}(\tau )$ can be easily compared to one another. 

In this work, protein concentration in microwell is non-dimensionalized by $K_{D}$, and is defined as $C^{*}=C/K_{D}$, and $C_{0}^{*}=C_{0}/K_{D}$. Protein concentration at surface of glass slide is non-dimensionalized by $C_{0}^{*}$ and is defined as ${\text{Γ}}^{'*}={\text{Γ}}^{'}/C_{0}^{*}$. Distance from surface of the single cell *r* is non-dimensionalized by ${\text{Γ}}_{\infty }/K_{D}$ and is specified as $r^{*}=rK_{D}/{\text{Γ}}_{\infty }$. The boundary condition at the sublayer of glass slide is expressed by following Equation (5), correlating the protein consumption flux at the sublayer of glass slide to the kinetic adsorption [23]:

(6) $${\left(\partial C^{*}/\partial r^{*}\right)}_{R_{0}^{*}=K_{D}R_{0}/{\text{Γ}}_{\infty }}=\partial {\text{Γ}}^{'}/\partial \tau =k_{a}\left(C_{R_{0}}^{*}\left(1-{\text{Γ}}^{"}\right)-{\text{Γ}}^{'}\right)$$

where *R*_0_ represents the distance of the surface of glass slide from the surface of the single cell.

The following procedures [16,17] were used to obtain numerical solutions for above equations. For clean glass slide capture, the initial surface coverage ${\text{Γ}}^{'}(\tau )$ is small, and the kinetic Equation (4) is linear ($d{\text{Γ}}^{'}/d\tau =k_{a}\left(C_{R0}^{*}-{\text{Γ}}^{'}\right)$). An analytical solution of Equation (4) was developed. A small Δτ was chosen as the time step. The kinetic binding at the first few (i-1) time steps ${\text{Γ}}^{'}(\tau_{i})$ ($\tau_{i}=i\Delta \tau$) were calculated using above analytical solutions. For the next (i th) step, (a) the i-2 and i-1 values of $C_{R0}^{*}$ and ${\text{Γ}}^{'}(\tau )$ solutions were used to obtain an extrapolated value for $C_{R0}^{*}\left(\tau_{i}\right)$ and ${\text{Γ}}^{'}(\tau_{i})$; (b) Equation (4) was used to compute ${\left(d{\text{Γ}}^{'}/d\tau \right)}_{i}$, the derivative at step i, from the values of $C_{R0}^{*}\left(\tau_{i}\right)$ and ${\text{Γ}}^{'}(\tau_{i})$ obtained in (a); a corrected estimate of ${\text{Γ}}^{'}(\tau_{i})$ was obtained from the value of the derivative at step i; (c) Equation (5) was applied to obtain a corrected value for $C_{R0}^{*}\left(\tau_{i}\right)$ with the (d) comparison of the corrected values with the extrapolated estimate from step (a); and iteration of steps (b) and (c) to generate further corrected values till reaching a prescribed tolerance.

## 2. Experimental Section

### 2.1. Recombinant Protein, Antibody and Antibody Conjugations

Recombinant interleukin 17 (IL17) and monoclonal antibodies used to capture IL17 were purchased from eBioscience (Minneapolis, MN, USA). Affinity-purified polyclonal antibodies for detecting IL17 (eBioscience) were labeled by conjugating the antibodies with NHSester activated fluorescent dyes, and purified by spin column (Invitrogen, Carlsbad, CA, USA). The average degree of labeling using the commercial kits was 3–4 dyes per antibody. 

### 2.2. Preparing Poly-Lysine Glass Slides

Poly-L-lysine slides were prepared based on published protocols available online (http://cat.ucsf.edu/pdfs/PolylysineSlides.pdf). Briefly, 3″ × 1″ glass slides (Corning, Lowell, MA, USA) were cleaned in 2.5 M NaOH in 60% ethanol for 2 h, and thoroughly washed with deionized (DI) water. Cleaned slides were submerged in 0.001% poly-L-lysine solution (diluted in 0.1 × PBS) for 1 h, further washed with DI water, dried, and stored in a desiccator until use.

### 2.3. Immobilization of Capture Antibody on Poly-Lysine Glass Slides

Capture antibody was immobilized on glass slides functionalized with poly-lysine for 2 h at room temperature (25 μg/mL concentration in Borate buffer comprising 50 mM sodium borate, 8 mM sucrose, and 50 mM NaCl (pH 9.0)). The slides were blocked with BSA (1% w/v in PBS) for 1 h at room temperature, washed three times with PBS, dipped in DI water, and spun dry.

### 2.4. Staining Captured Proteins on Reference Slides 

A capture antibody (50 μg/mL) was spotted on the surface of poly-L-lysine slides (1 μL/spot) and incubated for 1 h at room temperature. After blocking and washing the surface, the glass slide was placed in a 96-well Microplate Microarray Hardware (MMH96, ArrayIt, Sunnyvale, CA, USA). Then, 100 μL of recombinant IL17 (10 ng/mL) was added on each spot. The serial dilution was conducted to obtain 4–5 spots on the same glass slide with different doses. After a 2 h incubation at 37 °C, the slide was washed. A fluorescently labeled detection antibody (2 μg/mL) was applied for 30–60 min in the dark at room temperature. After incubation, the slide was washed with 0.05% Tween in PBS for three times, PBS three times, dipped in DI water, and spun dry. Images of the microarrays were collected by laser-based microarray scanners (Genepix 4000B and 4200AL, Molecular Devices, Sunnyvale, CA, USA), and analyzed using the accompanying software (Genepix Pro, Molecular Devices, Sunnyvale, CA, USA) to quantify the mean fluorescent intensities (MFIs) of each spot within the array.

## 3. Results and Discussions

### 3.1. Characteristic Diffusion Time Scale $\tau_{D}$ and Kinetic Time Scale $\tau_{K}$

The dynamic protein adsorption process in microengraving immunoassay includes protein secretion from a single cell, molecular diffusion and kinetic binding onto the glass slide surface. Two time scales characterize this process. The first, $\tau_{D}$, is the time required for molecular diffusion of proteins in the microwell to establish an equilibrium monolayer assuming infinitely fast surface binding kinetics. The second, $\tau_{K}$, is the kinetic time necessary for protein in the sublayer to bind onto the glass slide surface in the absence of molecular diffusion. The two characteristic diffusion and kinetic time scales $\tau_{D}$ and $\tau_{K}$ were calculated for different conditions using the scaling method [16,17,18,22] as detailed in Table 1.

**Table 1:** Characteristic diffusion time scale $\tau_{D}$ and kinetic time scale $\tau_{K}$ at different conditions.

	*k_on_* (M^−1^·s^−1^)	*k_off_* (s^−1^)	*K_D_* (M)	ϕ (molecule/s)	$\tau_{D}$ (s)	$\tau_{K}$ (s)	$k_{a}$
C^*^_0_ = 0.313	1.15 × 10^4^	0.0001	8.7 × 10^−9^	1	14.5	7620	1.45 × 10^−3^
	1.15 × 10^4^	0.001	8.7 × 10^−8^	10	14.5	762.0	1.45 × 10^−2^
	1.15 × 10^4^	0.01	8.7 × 10^−7^	100	14.5	76.2	0.145
	1.15 × 10^4^	0.1	8.7 × 10^−6^	1000	14.5	7.62	1.45
C^*^_0_ = 3.13	1.15 × 10^5^	0.0001	8.7 × 10^−1^°	1	14.5	2420	1.45 × 10^−3^
	1.15 × 10^5^	0.001	8.7 × 10^−9^	10	14.5	242.0	1.45 × 10^−2^
	1.15 × 10^5^	0.01	8.7 × 10^−8^	100	14.5	24.2	0.145
	1.15 × 10^5^	0.1	8.7 × 10^−7^	1000	14.5	2.42	1.45
C^*^_0_ = 31.3	1.15 × 10^6^	0.0001	8.7 × 10^−11^	1	14.5	310	1.45 × 10^−3^
	1.15 × 10^6^	0.001	8.7 × 10^−1^°	10	14.5	31.0	1.45 × 10^−2^
	1.15 × 10^6^	0.01	8.7 × 10^−9^	100	14.5	3.1	0.145
	1.15 × 10^6^	0.1	8.7 × 10^−8^	1000	14.5	0.31	1.45

In Table 1, the parameters of the cell’s biophysical properties and related with proteins were determined from reported values in the literature [21]. In this work, the diameter of a single lymphocyte was fixed as 5 μm. The diffusion coefficient D was defined as $1.4\times 10^{-10}{\text{ m}}^{\text{2}}\text{/s}$. The secretion rate of ϕ was varied from 1 molecule/s to 1000 molecules/s, which cover the low secretion rates (<molecules/s) from primary cytokine or chemokine secreting cells to high secretion rates (in the order of magnitude of 1000 molecules/s) from activated B lymphocytes including plasma cells and plasmablasts as well as optimized cell lines used in biomanufacturing such as CHO cells [24,25]. The maximum surface concentration ${\text{Γ}}_{\infty }$ was calculated as $8.47\times 10^{-9}{\text{ mole/m}}^{\text{2}}$ [21]. In this work, the $C_{0}^{*}$ was calculated at typical microengraving incubation time of 2 h unless specified. Associate constant $k_{on}$ and dissociate constant $k_{off}$ were chosen therefore the values of $K_{D}$ (the ratio of $k_{off}$ to $k_{on}$ physically represents the equilibrium dissociation constant between the antibody and its antigen), in the range of the representative values of mouse monoclonal antibodies [21,26,27]. The values of these parameters was chosen based on experimental measurements [22] and also varied systematically with increment of 10-, 100- and/or 1000- fold changes. The length of protein’s molecular diffusion is constrained by the distance between the surface of single cell and the glass slide surface as 45 um. This gives the characteristic time scale for diffusion $\tau_{D}$ of 14.5 s. $\tau_{D}$ will be a useful lower bound for the protein adsorption, since the transport dynamics cannot be faster than the diffusion-controlled cases. $C_{0}^{*}$ is mainly determined by the secretion rate and $K_{D}$. At certain secretion rate ϕ, an increase of $C_{0}^{*}$ results in decrease of kinetic time scales of $\tau_{K}$ as detailed in Table 1. At fixed $C_{0}^{*}$, kinetic time scale $\tau_{K}$ becomes 10 times smaller when the secretion rate ϕ is increased 10 times higher in the range of 1–1000 molecules/s. $\tau_{K}$ has a wide range of values from 0.31 s to 7620 s. Higher kinetic time scale $\tau_{K}$ requires longer time for proteins adsorption to be equilibrated on the glass slide surface. 

### 3.2. Concentration of Protein Captured on Glass Slide Surface as a Function of Time ${\text{Γ}}^{'}(\tau )$

**Figure 2:**
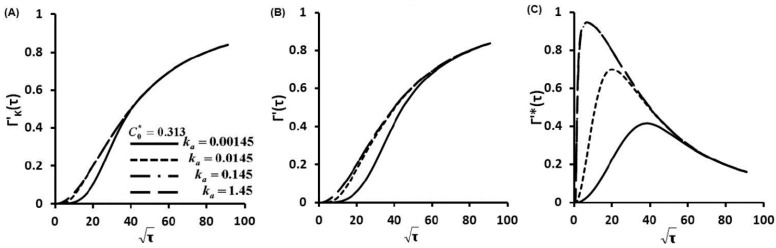
Concentration of protein captured on glass slide surface as a function of time ${\text{Γ}}^{'}\left(\tau \right)$ for $C_{0}^{*}=0.313$, (**A**) kinetic controlled, (**B**) ${\text{Γ}}^{'}\left(\tau \right)$ and (**C**) ${\text{Γ}}^{'*}(\tau )$ at finite diffusion-kinetic cases.

**Figure 3:**
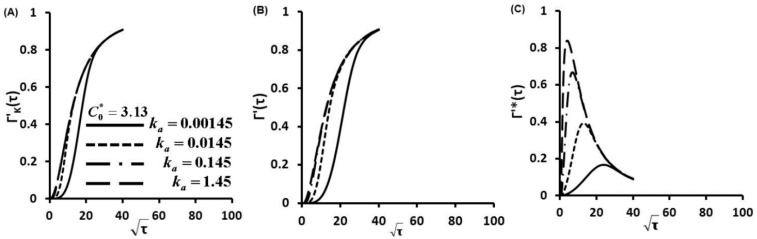
Concentration of protein captured on glass slide surface as a function of time ${\text{Γ}}^{'}\left(\tau \right)$ for $C_{0}^{*}=3.13$, (**A**) kinetic limited, (**B**) ${\text{Γ}}^{'}\left(\tau \right)$ and (**C**) ${\text{Γ}}^{'*}(\tau )$ at finite diffusion-kinetic cases.

**Figure 4:**
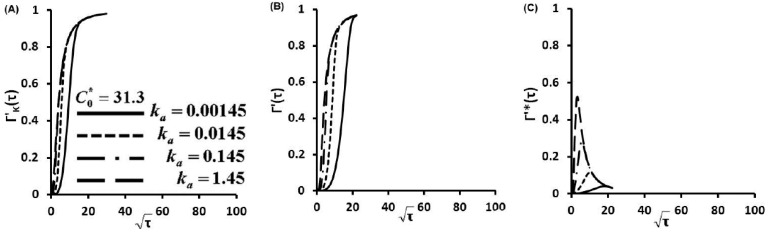
Concentration of protein captured on glass slide surface as a function of time ${\text{Γ}}^{'}\left(\tau \right)$ for $C_{0}^{*}=31.3$, (**A**) kinetic limited, (**B**) ${\text{Γ}}^{'}\left(\tau \right)$ and (**C**) ${\text{Γ}}^{'*}(\tau )$ at finite diffusion-kinetic cases.

${\text{Γ}}^{'}(\tau )$ describes the nondimensionalized concentration of proteins captured on glass slide surface as a function of dimensionless time τ. ${\text{Γ}}^{'}(\tau )$ was calculated as a function of $k_{a}$ (also a function of $K_{D}$) at $C_{0}^{*}=0.313$ for diffusion-controlled cases (Figure S1A), kinetic controlled cases (Figure 2A) and diffusion-kinetic mixed cases (Figure 2B,C). These figures represent a thousand-fold increase in the protein secretion rate ϕ as well as binding affinity $K_{D}$. Similar simulations were carried out for one hundred times increment of $C_{0}^{*}$ at $C_{0}^{*}=3.13$ (Figure 3) and $C_{0}^{*}=31.3$ (Figure 4). 

Equation (2) defines adsorption dynamic profiles under diffusion controlled mechanism, in which the amount of captured protein is proportional to the product of the square root of time and the bulk concentration which equals the protein concentration on the surface of the single cell. As we know that $C_{0}\propto N$. N=ϕt, therefore, *C*_0_ is proportional to *φ*:

(7) $${\text{Γ}}_{D}\left(t\right)\propto \phi \sqrt{Dt^{3}/\pi }$$

Equation (7) states that the amount of captured protein is mainly proportional to the secretion rate. Diffusion controlled profiles (Figure S1A) show the rapid increase of ${\text{Γ}}^{'}(\tau )$ with $\sqrt{\tau }$. From Figure S1A, we can also see that the adsorption dynamics is mainly influenced by the secretion rate ϕ and is independent of kinetic parameters of $k_{on}$, and $k_{off}$. In which the increases of secretion rate ϕ are indicated by the increases of *k*_a_ (from right to left in Figure S1). Diffusion controlled profiles provide the upper limits of adsorption dynamics. Linear trends are clearly demonstrated in these diffusion controlled cases (Figures S1A). 

The Langmuir kinetic equation (Equation (3)) gives the kinetic limited profiles (Figure 2A, Figure 3A and Figure 4A). These profiles show that protein adsorptions at kinetic limited cases are much slower than those of diffusion limited cases (Figure S1A) since protein molecules have to overcome a kinetic barrier to adsorb onto the glass slide surface.

**Figure 5:**
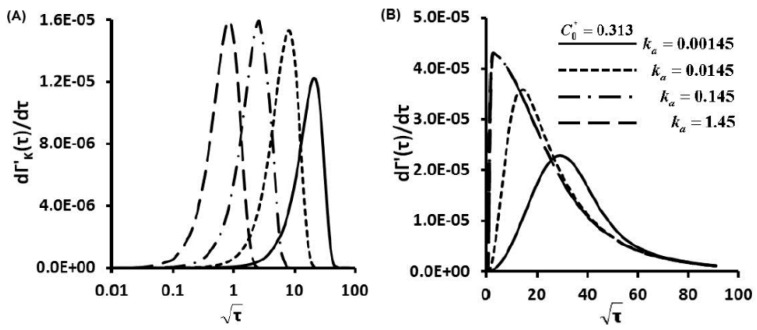
Protein Adsorption Rates as a Function of Time $d{\text{Γ}}^{'}/d\tau$ for $C_{0}^{*}=0.313$, (**A**) kinetic controlled; (**B**) finite diffusion-kinetic cases.

**Figure 6:**
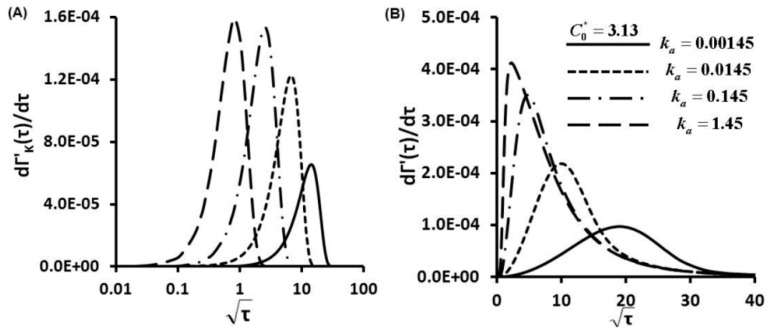
Protein Adsorption Rates as a Function of Time $d{\text{Γ}}^{'}/d\tau$ for $C_{0}^{*}=3.13$, (**A**) kinetic limited; (**B**) finite diffusion-kinetic cases.

**Figure 7:**
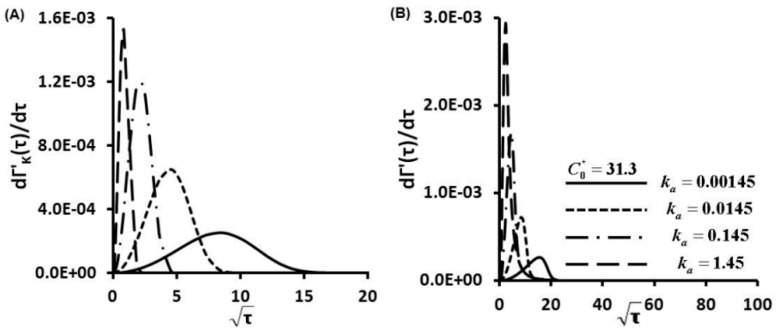
Protein Adsorption Rates as a Function of Time $d{\text{Γ}}^{'}/d\tau$ for $C_{0}^{*}=31.3$, (**A**) kinetic limited; (**B**) finite diffusion-kinetic cases.

The linear correlations between the nondimentional ${\text{Γ}}^{'*}(\tau )$ and square root of non-dimensional time $\sqrt{\tau }$) before curve's peaks are clearly shown in Figure 2C, Figure 3C and Figure 4C. These linear trends indicate that molecular diffusion play important roles in the early stage of protein adsorption dynamics. Then, kinetic factors take effect in the protein adsorption process. Kinetic barriers slow down the protein adsorption, reduce protein adsorption rates and result in the turning of the linear relationship in protein adsorption dynamics (Figure 2C, Figure 3C and Figure 4C). At fixed $C_{0}^{*}$, an increase of secretion rates increases $k_{a}$ (also $K_{D}$). This results in the earlier turning of the linear correlation (Figure 5B, Figure 6B and Figure 7B), which suggests that protein adsorption mechanism is switched from a diffusion controlled to a kinetic controlled mechanism. This observation is also consistent with other studies [28,29,30,31]. Quicker shifting to kinetic controlled mechanism at higher secretion rates (higher $k_{a}$ and also $K_{D}$) suggests a faster shift in the transport mechanism. Similar dynamic pathway of protein adsorption is observed with notably fast rates and rapid shifts in transport mechanisms when $C_{0}^{*}$ is increased a hundred times from 0.313 to 31.3. From Figure 2C to Figure 4C, the $C_{0}^{*}$ increases 100 fold from 0.313 to 31.3. The maximum surface concentration ${\text{Γ}}_{\infty }$ was defined by the maximum binding cites of capture antibody (Ab). In our study, ${\text{Γ}}_{\infty }$ was the same since we coated the same concentration of capture Ab. Therefore, the maximum values of ${\text{Γ}}^{'*}(\tau )$ decrease with the 100 fold increase of $C_{0}^{*}$ as shown from Figure 2C to Figure 4C. 

Torres *et al.* used a commercial software (COMSOL Multiphysics 3.3) and simulated the protein adsorption dynamics in both open and close systems [32]. Surface concentrations are not saturated in several situations of the open system. There are several different situations occurred during the microengraving immunoassay. First, the maximum surface concentration Γ_∞_ (same as the maximum protein binding sites) is determined by the concentration of capture antibody coated on the poly-lysine glass slide. Since we used the same concentration of capture antibody, the maximum surface concentration Γ_∞_ stays the same. Second, the adsorption of protein onto the glass slide is similar as the binding between the antigen and its antibody, it is very specific. As long as there is free binding site, the protein will bind onto the capture antibody. This will make the surface concentration approach the saturation at long enough time. Third, the bulk concentration is not a constant as used in the surfactant adsorption process. Protein secretion by a single cell is generally assumed as a continuous process [21,32,33] so the bulk concentration of protein $C_{0}^{*}\left(\tau \right)$ accumulate with time and become bigger and bigger (Figure S3). Therefore Γ_e_ can approach Γ_∞,_ or Γ_e_/ Γ_∞_ approaches 1 if C_0_/K_D_ is big enough (Equation (1)). 

### 3.3. Protein Adsorption Rate as a Function of Time $d{\text{Γ}}^{'}/d\tau$

The first order derivative of Equation (2) defines protein adsorption rates under a diffusion controlled mechanism. Straight lines are clearly shown in plots of protein adsorption rates $d{\text{Γ}}^{'}/d\tau$ as a function of square root of non-dimensional time $\sqrt{\tau }$) (Figure S1B). In diffusion controlled cases, increase of secretion rate accompanies increases of $k_{a}$ and $K_{D}$, and results in higher magnitudes of adsorption rates (Figure S1B). Maximum magnitudes of adsorption rates are mainly determined by the secretion rates and independent of $C_{0}^{*}$ (Figure S1B).

The first order derivative of the Langmuir kinetic equation (Equation (3)) gives the protein adsorption rates under a kinetic controlled mechanism (Figure 5A, Figure 6A and Figure 7A), in which different scales were used to provide maximum information. Maximum magnitudes of protein adsorption rates at kinetic limited cases are about several order magnitudes lower than those of diffusion limited cases (Figure S1B). Kinetic controlled profiles appear as Gaussian distributions not linear correlations between $d{\text{Γ}}^{'}/d\tau$ and $\sqrt{\tau }$. At $C_{0}^{*}=0.313$, increment of secretion rate increases $k_{a}$ and also $K_{D}$, and adsorption rate curves shift from right to left side with increase of maximum magnitudes (Figure 5B). At fixed $k_{a}$, increased values of $C_{0}^{*}$ to 3.13 and 31.3 also increase ten to hundred times the maximum magnitudes of adsorption rates (Figure 6B and Figure 7B).

At the early stage, the protein adsorption rate $d{\text{Γ}}^{'}/d\tau$ increases with the square root of nondimensional time $\sqrt{\tau }$) in an approximately linear correlation till reaching peak values (Figure 5B, Figure 6B and Figure 7B). Protein adsorption behaviors within this stage show similar trends in diffusion controlled cases. The diffusion mechanism is dominant at this stage, then transport mechanism switches over to kinetic controlled when maximum adsorption rates are attained. At fixed $C_{0}^{*}$, the highest protein adsorption rate $d{\text{Γ}}^{'}/dt$ is achieved at the highest secretion rate (the highest $k_{a}$ and also the highest $K_{D}$). Then, protein adsorption rates decay in an exponential manner till reaching equilibrium values. The differences between the maximum $d{\text{Γ}}^{'}/d\tau$ become more notable with increase of $C_{0}^{*}$ as illustrated from Figure 5B to Figure 7B. Transport mechanism in protein adsorption dynamics shifts earlier at higher $K_{D}$ (also higher secretion rates), as indicated by decreased adsorption rates after reaching the turning points (peaks of Figure 5B, Figure 6B and Figure 7B).

For finite diffusion-kinetic protein adsorption, both molecular diffusion and kinetic adsorption take effects. Protein adsorption rates $d{\text{Γ}}^{'}/d\tau$ are combination of molecular diffusion and kinetic adsorption contributions. Magnitudes of protein adsorption rates $d{\text{Γ}}^{'}/d\tau$ are in the ranges of upper limits of diffusion limited and lower limits of kinetic limited cases. The trends of protein adsorption rates $d{\text{Γ}}^{'}/d\tau$ are also kinds of combinations of diffusion limited and kinetic limited cases. Kinetic effects make more contributions to the protein adsorption dynamics with increase of $C_{0}^{*}$. The shapes of protein adsorption rates $d{\text{Γ}}^{'}/d\tau$ become more similar as those of kinetic limited with increases of $C_{0}^{*}$ after reaching peak values. These results further support that transport mechanism of dynamic protein adsorption shifts from molecular diffusion controlled to kinetic influenced with increases of $C_{0}^{*}$.

### 3.4. Concentration of Protein in the Solution and at Sublayer as a Function of Time C(r,t) and $C_{R0}^{'}\left(t\right)$

Concentration of protein at sublayer $C_{R0}^{'}\left(t\right)$ is normalized with protein concentration at the single cell’s surface $C_{0}$. Magnitudes of $C_{R0}^{'}\left(t\right)$ increase with time and approach to 1 eventually. At fixed $C_{0}^{*}$, magnitudes of $C_{R0}^{'}\left(t\right)$ are also influenced by the secretion rates. The higher secretion rates, the higher $K_{D}$. This also results in higher $C_{R0}^{'}\left(t\right)$, and faster approaching of the $C_{R0}^{'}\left(t\right)$ to 1. The differences within $C_{R0}^{'}\left(t\right)$ at different secretion rates become more notable with the increase of $C_{0}^{*}$ from 0.313 to 31.3 (Figure S2).

For concentration distribution curve of $C^{*}\left(r,t\right)$ as a function of distance from the surface of the single cell, sharp exponential decay curves are observed at initial stages (data not shown). These curves quickly develop into linear decreasing lines during the microengraving process. Less sharp exponential decay curves are observed eventually. $C_{0}^{*}$ (Figure S3) is determined crucially by the secretion rate. $C_{R0}^{'}\left(t\right)$ is determined by both the secretion rate *φ* and the kinetic parameter $K_{D}$. As we knew, the bulk concentration $C_{0}$ defined as protein concentration on the surface of the single cell at certain incubation time is proportional to the total proteins secreted from the single cell, *i.e.*, $C_{0}\propto N$. N=ϕt, and $C_{0}^{*}=C_{0}/K_{D}$. We can infer that $C_{0}^{*}\propto \phi /K_{D}$. From Table 1, we can see that the ratios of ϕ to K_D_ remain the same for all the cases at a fixed value of $C_{0}^{*}$. Consistent results are demonstrated for a wide ranges of $C_{0}^{*}$ and $k_{a}$.

### 3.5. Protein Adsorption Isotherms

**Figure 8:**
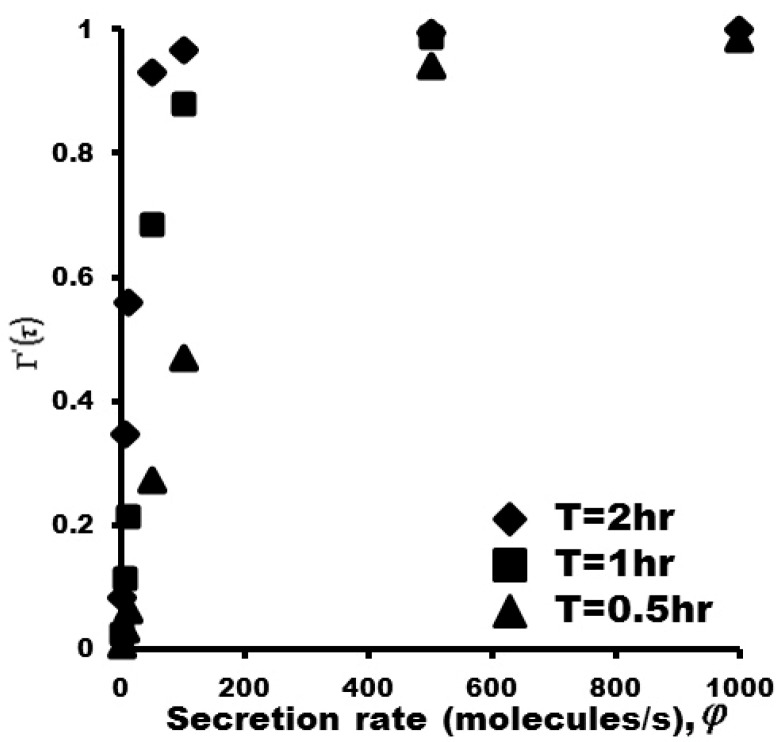
Adsorption isotherms (*K_D_* = 8.7 × 10^−9^ M) at different incubation time of 2 h, 1 h, and 0.5 h.

The correlation between the concentration of protein captured on the glass slide ${\text{Γ}}^{'}(\tau )$ and the protein concentration on the surface of the single cell *C*_0_, which are calculated at a certain incubation time of the microengraving immunoassay, gives protein adsorption isotherms. As we knew that the bulk concentration $C_{0}$ defined as protein concentration on the surface of the single cell at certain incubation time is proportional to the total proteins secreted from the single cell, *i.e.*, $C_{0}\propto N$. N=ϕt. *C*_0_ is proportional to *φ*. Therefore, the adsorption isotherm of ${\text{Γ}}^{'}(\tau )$ *vs.* *φ* can be constructed. Kinetic parameters of capture Abs are fixed in experiments. In this work, adsorption isotherms of ${\text{Γ}}^{'}(\tau )$ were calculated as a function of secretion rate *φ* at fixed K_D_ and at different incubation time including 2 h, 1 h and 0.5 h (Figure 8) covering the protein secretion rate from 1–10,000 molecules/s. The zoomed part of range from 1 to 100 molecules/s is shown in Figure S4. 

**Figure 9:**
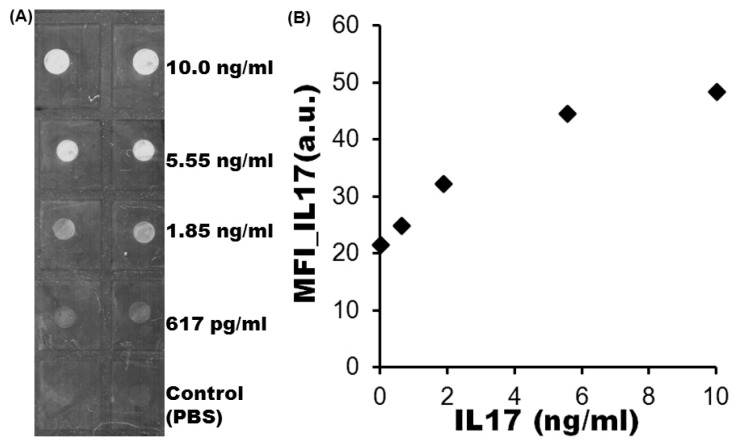
Experimentally obtained adsorption isotherm of interleukin (IL17). (**A**) image of spot assay, (**B**) experimental adsorption isotherm of IL17.

Parameter $C_{0}^{*}$ is proportional to the ratio of protein secretion rate φ at certain incubation time to binding affinity $K_{D}$. Under defined values of $K_{D}$, the value of $C_{0}^{*}$ is determined exclusively by protein secretion rate *φ*. The concentration of protein captured on the glass slide ${\text{Γ}}^{'}(\tau )$ of y axis of theoretical adsorption isotherm (Figure 8) is proportional to MFIs of y axis of experimental adsorption isotherm (Figure 9B). The secretion rate *φ* of x axis of theoretical adsorption isotherm (Figure 8) is proportional to concentration of recombinant IL17 of x axis of experimental adsorption isotherm (Figure 9B). The consistence of theoretical adsorption isotherms (Figure 8) with the experimental result (Figure 9B) indicates that protein secretion rates from a single cell can be quantified by the microengraving immunoassay.

## 4. Conclusions

Time evolutions of protein concentration captured on the glass slide surface and in the confined volume of microwells have been investigated with one thousand-fold changes of secretion rates ϕ and kinetic associate and dissociate constants $k_{on}$ and $k_{off}$. Controlling mechanisms for protein adsorption dynamics change as functions of secretion rates, binding affinity $K_{D}$ and bulk concentration $C_{0}^{*}$ from diffusion to mixed kinetic-diffusion control. At the initial stage, protein bulk concentration is low, and the influence of molecular diffusion is more important in protein adsorption dynamics. With increase of bulk concentration of $C_{0}^{*}$, kinetic control takes effect. The dynamic protein adsorption process shifts from diffusion controlled to kinetic influenced in the transport mechanism.

Theoretical adsorption isotherms are obtained by the employed numerical method considering the molecular diffusion of proteins at the sublayer of the glass slide in the microwell, combined with the adsorption kinetics at the sublayer of the glass slide at different $C_{0}^{*}$ and $k_{a}$ (also different $K_{D}$). The theoretical adsorption isotherms follow a similar trend as the experimentally obtained calibration curve, in which the amount of protein from individual cells captured on a binding surface increases monotonically with secretion rate. Therefore the microengraving immunoassay can be used to quantify the protein secretion rates from single cells, and determine the distribution of secretion rates for proteins of interest among single cells within a cell population. 

Understanding of protein adsorption dynamics helps us to quantify protein secretion rates for ~10^4^–10^5^ single primary human lymphocytes in parallel. This quantitative analysis of secretion rates allow us to screen antigen-specific cells with highest secretion rate, retrieve single cells of interest for clonal expansion and reveal cellular heterogeneity within a cell sample. 

## Supplementary Files

Supplementary File 1

## References

[1] Harari A., Vallelian F., Meylan P.R., Pantaleo G. (2005). Functional heterogeneity of memory CD4 T cell responses in different conditions of antigen exposure and persistence. J. Immunol..

[2] Kindt T.J., Goldsby R.A., Osborne B.A. (2007). Immunology.

[3] Stanimirova R.D., Marinova K.G., Danov K.D., Kralchevsky P.A., Basheva E.S., Stoyanov S.D., Pelan E.G. (2014). Competitive Adsorption of the Protein Hydrophobin and an Ionic Surfactant: Parallel *vs* Sequential Adsorption and Dilatational Rheology. Colloid Surf. A.

[4] Turcanu V., Williams N.A. (2001). Cell identification and isolation on the basis of cytokine secretion: A novel tool for investigating immune responses. Nat. Med..

[5] Bevan M.J. (2004). Helping the CD8(+) T-cell response. Nat. Rev. Immunol..

[6] Darrah P.A., Patel D.T., de Luca P.M., Lindsay R.W., Davey D.F., Flynn B.J., Hoff S.T., Andersen P., Reed S.G., Morris S.L. (2007). Multifunctional TH1 cells define a correlate of vaccine-mediated protection against Leishmania major. Nat. Med..

[7] Kannanganat S., Ibegbu C., Chennareddi L., Robinson H.L., Amara R.R. (2007). Multiple-cytokine-producing antiviral CD4 T cells are functionally superior to single-cytokine-producing cells. J. Virol..

[8] Román E., Miller E., Harmsen A., Wiley J., von Andrian U.H., Huston G., Swain S.L. (2002). CD4 effector T cell subsets in the response to influenza: Heterogeneity, migration, and function. J. Exp. Med..

[9] Duvall M.G., Precopio M.L., Ambrozak D.A., Jaye A., McMichael A.J., Whittle H.C., Roederer M., Rowland-Jones S.L., Koup R.A. (2008). Polyfunctional T cell responses are a hallmark of HIV-2 infection. Eur. J. Immunol..

[10] Bradshaw E.M., Kent S.C., Tripuraneni V., Orban T., Ploegh H.L., Hafler D.A., Love J.C. (2008). Concurrent detection of secreted products from human lymphocytes by microengraving: Cytokines and antigen-reactive antibodies. Clin. Immunol..

[11] Love J.C., Ronan J.L., Grotenbreg G.M., van der Veen A.G., Ploegh H.L. (2006). A microengraving method for rapid selection of single cells producing antigen-specific antibodies. Nat. Biotechnol..

[12] Story C.M., Papa E., Hu C.C., Ronan J.L., Herlihy K., Ploegh H.L., Love J.C. (2008). Profiling antibody responses by multiparametric analysis of primary B cells. Proc. Natl. Acad. Sci. USA.

[13] Song Q., Han Q., Bradshaw E.M., Kent S.C., Raddassi K., Nilsson B., Nepom G.T., Hafler D.A., Love J.C. (2010). On-Chip Activation and Subsequent Detection of Individual Antigen-Specific T Cells. Anal. Chem..

[14] Ogunniyi A.O., Story C.M., Papa E., Guillen E., Love J.C. (2009). Screening individual hybridomas by microengraving to discover monoclonal antibodies. Nat. Protoc..

[15] Jin A., Ozawa T., Tajiri K., Obata T., Kondo S., Kinoshita K., Kadowaki S., Takahashi K., Sugiyama T., Kishi H. (2009). A rapid and efficient single-cell manipulation method for screening antigen-specific antibody-secreting cells from human peripheral blood. Nat. Med..

[16] Song Q., Couzis A., Somasundaran P., Maldarelli C. (2006). A transport model for the adsorption of surfactant from micelle solutions onto a clean air/water interface in the limit of rapid aggregate disassembly relative to diffusion and supporting dynamic tension experiments. Colloid Surf. A Physicochem. Eng. Aspects.

[17] Song Q., Yuan M. (2011). Visualizing an adsorption model for surfactant transport from micellar solutions to a clean air/water interface by fluorescence microscope. J. Colloid Interface Sci..

[18] Pan R., Green J., Maldarelli C. (1998). Theory and Experiment on the Measurement of Kinetic Rate Constants for Surfactant Exchange at an Air/Water Interface. J. Colloid Interface Sci..

[19] Eastoe J., Dalton J.S. (2000). Dynamic surface tension and adsorption mechanisms of surfactants at the air-water interface. Adv. Colloid Interface Sci..

[20] Chang C.-H., Franses E.I. (1995). Adsorption dynamics of surfactants at the air/water interface: a critical review of mathematical models, data, and mechanisms. Colloid Surface A Physicochem. Eng. Aspects.

[21] Frykman S., Srienc F. (1998). Quantitating secretion rates of individual cells: design of secretion assays. Biotechnol. Bioeng..

[22] Ferri J.K., Stebe K.J. (2000). Which surfactants reduce surface tension faster? A scaling argument for diffusion-controlled adsorption. Adv. Colloid Interface Sci..

[23] Lionello A., Josserand J., Jensen H., Girault H.H. (2005). Protein adsorption in static microsystems: Effect of the surface to volume ratio. Lab Chip.

[24] Charlet M., Kromenaker S.J., Srienc F. (1995). Surface IgG content of murine hybridomas: Direct evidence for variation of antibody secretion rates during the cell cycle. Biotechnol. Bioeng..

[25] Henn A.D., Rebhahn J., Brown M.A., Murphy A.J., Coca M.N., Hyrien O., Pellegrin T., Mosmann T., Zand M.S. (2009). Modulation of single-cell IgG secretion frequency and rates in human memory B cells by CpG DNA, CD40L, IL-21, and cell division. J. Immunol..

[26] Day Y.S., Baird C.L., Rich R.L., Myszka D.G. (2002). Direct comparison of binding equilibrium, thermodynamic, and rate constants determined by surface- and solution-based biophysical methods. Protein Sci..

[27] Foote J., Eisen H.N. (1995). Kinetic and affinity limits on antibodies produced during immune responses. Proc. Natl. Acad. Sci. USA.

[28] Cho D., Narsimhan G., Franses E.I. (1997). Adsorption Dynamics of Native and Pentylated Bovine Serum Albumin at Air-Water Interfaces: Surface Concentration/ Surface Pressure Measurements. J. Colloid Interface Sci..

[29] Um S.U., Poptoshev E., Pugh R.J. (1997). Aqueous Solutions of Ethyl (Hydroxyethyl) Cellulose and Hydrophobic Modified Ethyl (Hydroxyethyl) Cellulose Polymer: Dynamic Surface Tension Measurements. J. Colloid Interface Sci..

[30] Ybert C., di Meglio J.M. (1998). Study of protein adsorption by dynamic surface tension measurements: Diffusive regime. Langmuir.

[31] Zhmud B.V., Poptoshev E., Pugh R.J. (1998). Role of Hydration and Conformational Changes in Adsorption Dynamics of Ethyl(Hydroxyethyl)cellulose at the Air/Solution Interface. Langmuir.

[32] Torres A.J., Hill A.S., Love J.C. (2014). Nanowell-based immunoassays for measuring single-cell secretion: characterization of transport and surface binding. Anal. Chem..

[33] Han Q., Bradshaw E.M., Nilsson B., Hafler D.A., Love J.C. (2010). Multidimensional analysis of the frequencies and rates of cytokine secretion from single cells by quantitative microengraving. Lab Chip.

