# Supplementary material for: Protein Adsorption in Microengraving Immunoassays
Source: Sensors (Basel). 2015 Oct 16;15(10):26236–50. doi: 10.3390/s151026236 (PMC4634505; doi:10.3390/s151026236)
Supplement: Supplementary File 1 [file sensors-15-26236-s001.pdf]

OPEN ACCESS

sensors

ISSN 1424-8220

www.mdpi.com/journal/sensors

Supplementary Information

Protein Adsorption in Microengraving Immunoassays. *Sensors* 2015, *15*, 26236-26250

Qing Song

Chemical and Biomolecular Engineering, New York University Polytechnic School of Engineering, 6 Metro Tech Center, Brooklyn, NY 11201, USA; E-Mail: qs299@nyu.edu; Tel.: +1-718-260-3863

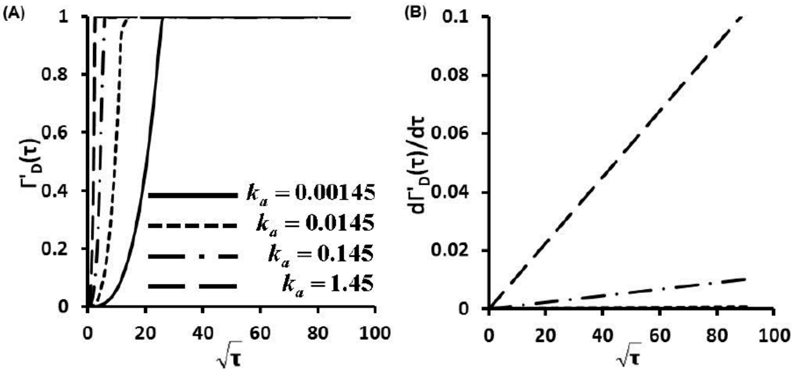

Figure S1. Diffusion controlled limits. (**A**) Concentration of protein captured on glass slide surface as a function of time $\Gamma'(\tau)$; (**B**) Protein adsorption rates as a function of time $d\Gamma'/d\tau$.

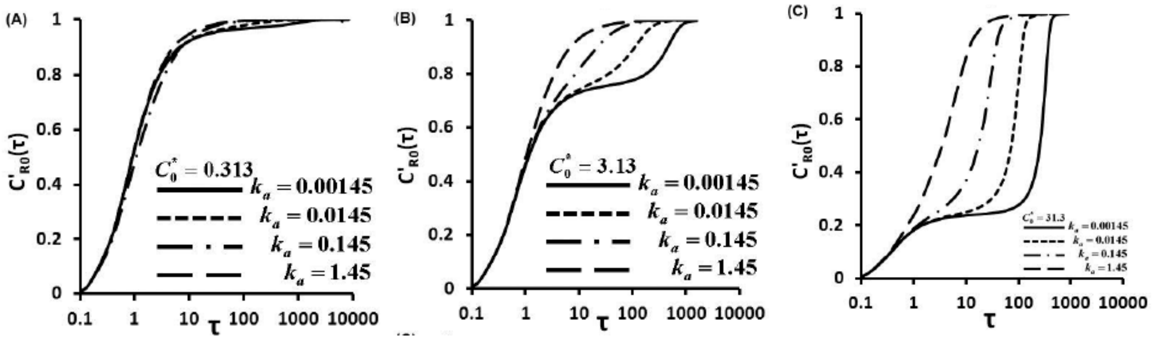

Figure S2. Concentration of protein at sublayer of the glass slide surface as a function of time $C'_{R_0}(\tau)$ at different $C_0^* = 0.313$ (**A**), $C_0^* = 3.13$ (**B**), and $C_0^* = 31.3$ (**C**).

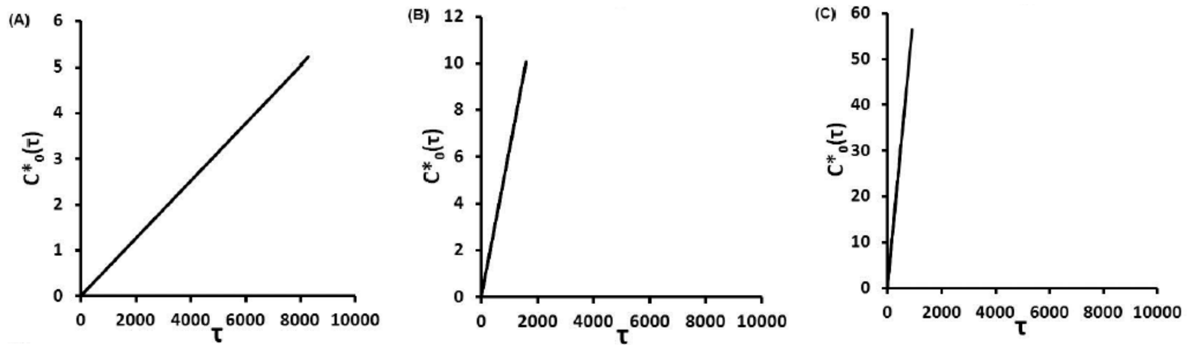

Figure S3. Concentration of protein at surface of the single cell as a function of time $C'_{R_0}(\tau)$ at different $C_0^* = 0.313$ (**A**), $C_0^* = 3.13$ (**B**), and $C_0^* = 31.3$ (**C**).

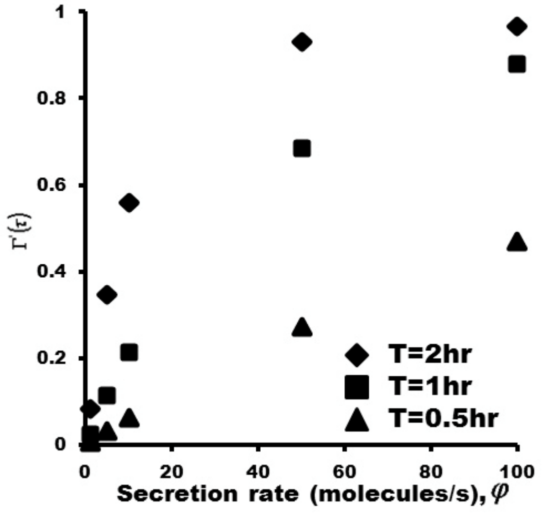

Figure S4. Adsorption isotherms ($K_D = 8.7 \times 10^{-9}$ M) at different incubation time of 2 h, 1 h, and 0.5 h in the range of 1–100 molecules/s.

© 2015 by the authors; licensee MDPI, Basel, Switzerland. This article is an open access article distributed under the terms and conditions of the Creative Commons Attribution license (http://creativecommons.org/licenses/by/4.0/).
